# Host-Parasite Interaction: Parasite-Derived and -Induced Proteases That Degrade Human Extracellular Matrix 

**DOI:** 10.1155/2012/748206

**Published:** 2012-06-26

**Authors:** Carolina Piña-Vázquez, Magda Reyes-López, Guillermo Ortíz-Estrada, Mireya de la Garza, Jesús Serrano-Luna

**Affiliations:** Departamento de Biología Celular, Centro de Investigación y de Estudios Avanzados del IPN, Aveinda Instituto Politécnico Nacional 2508, 07360 México, DF, Mexico

## Abstract

Parasitic protozoa are among the most important pathogens worldwide. Diseases such as malaria, leishmaniasis, amoebiasis, giardiasis, trichomoniasis, and trypanosomiasis affect millions of people. Humans are constantly threatened by infections caused by these pathogens. Parasites engage a plethora of surface and secreted molecules to attach to and enter mammalian cells. The secretion of lytic enzymes by parasites into host organs mediates critical interactions because of the invasion and destruction of interstitial tissues, enabling parasite migration to other sites within the hosts. Extracellular matrix is a complex, cross-linked structure that holds cells together in an organized assembly and that forms the basement membrane lining (basal lamina). The extracellular matrix represents a major barrier to parasites. Therefore, the evolution of mechanisms for connective-tissue degradation may be of great importance for parasite survival. Recent advances have been achieved in our understanding of the biochemistry and molecular biology of proteases from parasitic protozoa. The focus of this paper is to discuss the role of protozoan parasitic proteases in the degradation of host ECM proteins and the participation of these molecules as virulence factors. We divide the paper into two sections, extracellular and intracellular protozoa.

## 1. Introduction

The extracellular matrix (ECM) is the noncellular component present within all tissues and organs; it is produced mainly by a heterogeneous population of fibroblasts [1] and provides essential physical scaffolding for the cellular constituents as well as biochemical cues that are required for tissue morphogenesis, differentiation, and homeostasis. The ECM is composed of water, proteins, and polysaccharides; each tissue has an ECM with a unique and different composition and a distinct topology. Cell adhesion to the ECM is tissue specific and is mediated by ECM receptors, such as integrins, discoidin domain receptors, and syndecans. The ECM includes the interstitial matrix and the basement membrane, of which the interstitial matrix is present between cells, whereas the basement membrane is a thin, sheet-like deposition of ECM that surrounds cells (e.g., muscle cells) or underlies cells (e.g., epithelial cells). The basement membrane is composed of two layers: a basal lamina and a fibrillar reticular lamina [2, 3]. Adhesion mediates cytoskeletal coupling to the ECM and is involved in cell migration; the ECM is also a highly dynamic structure that is constantly being remodeled, both enzymatically and nonenzymatically, and its molecular components are subjected to various types and numbers of posttranslational modifications [4].

The ECM is composed of two main classes of macromolecules: proteoglycans (PGs) and fibrous proteins. PGs are composed of glycosaminoglycan (GAG) chains covalently linked to a specific protein core. PGs have been classified based on their core protein localization and GAG composition. The three main families are small leucine-rich proteoglycans (SLRPs), modular proteoglycans, and cell surface proteoglycans [5]. PGs occupy the majority of extracellular interstitial space within the tissue in the form of a hydrated gel [6]. PGs have a wide variety of functions that reflect their unique buffering, hydration, binding, and force-resistance properties. 

 The main fibrous ECM proteins are collagens, elastins, fibronectins, and laminin. Collagen is the most abundant fibrous protein within the ECM and constitutes up to 30% of the total protein mass of a multicellular animal. This protein constitutes the main structural element of connective tissues and also provides tensile strength, regulates cell adhesion, supports chemotaxis and migration, and directs tissue development [7]. Collagen associates with elastin, another major ECM fiber. Elastin provides recoil to tissues that undergo repeated stretch. A third fibrous protein, fibronectin (FN), is intimately involved in directing the organization of the interstitial ECM and also plays a crucial role in mediating cell attachment and function [8]. Additionally, FN is important for cell migration during development and has been implicated in cardiovascular disease and tumor metastasis [7]. Laminins and collagen type IV form independent networks that are connected by nidogen and perlecan [9].

## 2. Host-Parasite Relation

Every mammalian host is in constant danger of infection caused by pathogens, such as viruses, bacteria, fungi or parasites. Host defense against these pathogens requires a well-regulated inflammatory response marked by leukocyte migration into the site of infection, destruction of the microorganisms, resolution of inflammation, and, finally, healing and repair of the tissue architecture. Generally speaking, the relationship between host and parasite determines the outcome of the infection. Indeed, on an evolutionary scale, most parasites have developed adaptive mechanisms to evade host immune system responses. Some parasites evade the host's immune response by hiding intracellularly, such as *Toxoplasma* and *Plasmodium *species, and certain others evade the cell immune response completely including extracellular parasites such as *Entamoeba histolytica*, free-living amoebas, and *Trichomonas vaginalis*. 

Parasites engage a plethora of surface and secreted molecules to attach to and enter mammalian cells. Many of these molecules are involved in triggering specific signaling pathways, both in the parasite and the host cell, that are critical for parasite entry and survival. Several important advances have been achieved in identifying factors that are critical to parasite virulence and the pathogenesis of the diseases they cause. Among the most widely studied of these factors are parasite-derived proteases. Parasitic proteases can play a variety of roles in establishing, maintaining, and exacerbating an infection. Most of the human protozoan parasites invade, migrate, and reside within a variety of tissues and organs, whether they are intracellular or extracellular parasites. Interestingly for some parasites it has recently been reported the induction of ECM proteases in host cells. Connective tissue and basement membranes represent major barriers to parasite invasion, dissemination, and access to essential nutrients. Thus, mechanisms for connective tissue degradation might be critical for parasite survival. Therefore, we divide the paper into two sections discussing extracellular and intracellular protozoa.

## 3. Extracellular Protozoa

### 3.1. *Entamoeba histolytica*

*E. histolytica *is the causal agent of amoebiasis in humans and is responsible for an estimated 35 to 50 million cases of symptomatic diseases and approximately 100 000 deaths annually, mainly in the developing world [10]. Parasite cysts are transmitted through contaminated food and water. Parasite excystation in the small intestine produces eight trophozoites per cyst, which then colonize the large intestine [11]. Once *E. histolytica *trophozoites are normally established in the human colon, the infection has variable outcomes, including such manifestations as asymptomatic colonization, diarrhea, dysentery, invasive colitis, liver abscesses, or metastatic invasion. Parasite destruction of host cells appears to be the basis of disease; invasive disease pathologies, such as colitis and liver abscesses, are associated with tissue invasion and massive host tissue destruction [12]. For example, flask-shaped ulcers, a hallmark of amoebic colitis, is characterized by severe damage to enteric cells as well as the migration to the *lamina propria* and blood vessels [13]. It has been proposed that for the initial contact or adhesion, surface carbohydrates on the target cell are recognized by specific molecules (lectins). One of the more studied amoebic lectins is the Gal/GalNAc lectin, which mediates the binding to host carbohydrate determinants that contain galactose and/or N-acetyl-D-galactosamine (GalNAc) [11]. Other proteins also contribute to host cell binding on target cells [14]. The subsequent cell lysis occurs through the insertion of pore-forming proteins (amoebapores) into the host cell membranes [15], which allows a massive influx of extracellular Ca^+2^ [16] combined with the release of amoebic proteases at the site of contact, with the subsequent degradation of the substrate [17]. Once the targets are partially digested, the amoeba internalizes the cell debris and substrate fragments by phagocytosis [18]. In contrast, the interaction of trophozoites with extracellular matrix (ECM) components results in the proteolysis and destruction of the connective tissue [19]. *E*. *histolytica *possesses 50 cysteine protease (CP) genes [20]. These proteases have been demonstrated to act on a variety of host substrates *in vitro* [21–25]. At least some of these proteases are secreted, and a few have been characterized as surface localized; hence, they have the potential to contribute to host tissue breakdown *in vivo*. More than 80% of amoebic patients express antibodies to trophozoite CP [26].

An *in vitro* model was developed to analyze the interaction of *E. histolytica *trophozoites with ECM proteins [27]. The assays quantitatively monitored the adhesion of trophozoites to purified FN-covered surfaces and the breakdown of this protein under diverse experimental conditions. The data showed specificity in the binding and the occurrence of structural and biochemical events in the amoebas that participate in and promote the adhesion to the substrate and the later degradation. Similar results were obtained with laminin and Matrigel. A putative amoebic fibronectin receptor with a molecular weight of 37 kDa was found [27, 28]. Another protein of 140 kDa was found, with similarities to *β*-integrin family that together with the 37-kDa protein recognizes fibronectin and produces cytoskeletal changes in the amoebae [29]. The adhesion to fibronectin triggers proteolytic enzyme release, which facilitates the local degradation of the substrate [27, 28, 30]. Certain of these secreted proteases show similarities to cathepsin B [17] and might generate fragments with chemotactic and chemokinetic properties that are able to promote binding as well as locomotion of trophozoites [31]. 

**Figure 1:**
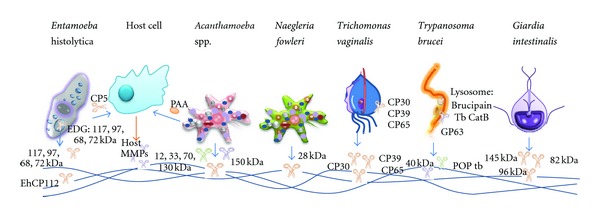
Extracellular parasite proteases. CPs: cysteine proteases, pink scissors; SPs: serine proteases, green scissors; MMPs: matrix metalloproteases, blue scissors; ECM extracellular matrix; EDG: electron-dense granules; POP: prolyl oligopeptidase; PAA: plasminogen activator activity; CatB: cathepsin B.

Collagen is a major component of the basal lamina and the ECM components of the intestine. There are three collagen-binding proteins described in *E. histolytica*, with molecular weights of 105, 56, and 30 kDa, that recognize mainly collagen type I; the 30 kDa protein has collagenolytic activity. Antibodies raised against the 30 kDa molecule inhibit the binding of trophozoites to collagen [32]. Several of the proteolytic activities related with ECM degradation are summarized in Table 1 and Figure 1.

An amoebic collagenase activity was first described by Muñoz et al. [57]; this study showed that this protein of *E. histolytica *was a membrane-bound enzyme that digests native collagen type I and type III at neutral pH and 37°C. The collagenase was more active against type I collagen. Three major fragments of 75, 50, and 25 kDa were obtained from collagen type I when this protein was incubated with *E. histolytica *trophozoites for 3 h. After this incubation period, smaller fragments of collagen were found, possibly due to the action of other proteolytic enzymes.

The collagenase activity was found mainly in electron-dense granules in *E. histolytica*. These granules were induced and secreted in response to the incubation of collagen type I with trophozoites of *E. histolytica in vitro* [58]. In another study, one specific collagenase activity with a molecular weight of 72 kDa was found in *E. histolytica* crude extracts [33]. This activity was found in electron-dense granules and could be related to the actin cytoskeleton function because one cytoskeleton-altered amoeba (BG-3) derived from the pathogenic HM1-IMSS strain had less collagenase activity [33]. 

Collagen type I incubation not only promotes collagenase activity but also increases the secretion of other proteases (mainly CP) [59], and, together with Ca^2+^, is able to induce the activation of several amoebic genes related to certain virulence factors, such as amoebapore C and cysteine protease 5, along with the stress-induced protein HSP70 and the ribosomal protein L27a [60]. In a recent study, Chávez Munguia et al. [35] demonstrated that electron-dense granules contain multiple cysteine protease activities.

There is evidence supporting the role of the extracellular cysteine proteases of *E*. *histolytica *as virulence factors. CP purified from axenized *E*. *histolytica* cleaves collagen, elastin, fibronectin, and laminin [21, 24, 34, 61–63]. CP-A5 and CP-B9 cysteine proteases possess gelatinase activity *in vitro *[34, 36] and may have a role during tissue invasion. Hou et al. [64] has shown that promature CP-A5 binds to colonocyte and triggers cytokine secretion. In a recent work [37] using 3D collagen matrix determined that amoebic CPs are responsible for the collagenase activity and that these enzymes have an important role during cell migration through a three-dimensional collagen scaffold. *E. histolytica *trophozoites combine cell shape deformation and protease activity in order to overcome physical constraints, suggesting that *E. histolytica*'s particular mode of migration explains its ability to overcome various environment constraints to rapidly invade human tissues. In this work, the authors also hypothesize that CP5 promotes inflammation and the secretion of host metalloproteases (MMP) that contribute to the ECM destruction.

Finally, the collagenolytic activity of *E. histolytica *has been correlated with its virulence when compared among different strains of *E*. *histolytica* [65–67] or with other virulence factors [68]. In all the studies, the more virulent strain always has the higher collagenolytic activity.

The study of *E. histolytica *proteases is an interesting field to be explored in the future as drug targets to inhibit the migration and invasion of this parasite. 

### 3.2. *Giardia intestinalis*

*G. intestinali*s (also known as *Giardia lamblia* and *Giardia duodenalis*) is a major contributor of diarrheal diseases in humans. The trophozoite is the disease-causing stage of the parasite [69]. An estimated 200 million people have symptomatic giardiasis worldwide, and children under 5 years are at particular risk [70]. Although *G. intestinalis* infection is not invasive, parasites adhere to the brush border microvilli lining on the small intestine surface, leading to a reduction in their height, accompanied by decreased expression and activity of several digestive enzymes located in the intestine. These alterations conduct to diarrhea and malabsorption syndrome [71, 72]. Adhesion to ECM could be important for colonization, since trophozoite attachment was demonstrated to be even more effective to type I collagen than to the apical surface of confluent Madin Darby canine kidney (MDCK) cells *in vitro* [73].

*Giardia* releases products that may contribute to pathogenesis, such as proteases, although they have not been well characterized yet [74, 75]. Little information is available regarding specific proteins against ECM. There are only three reports regarding the collagenolytic activity using zymograms (Table 1, Figure 1). Williams and Coombs [76] explored intracellular proteases present in lysates of trophozoites and observed collagen degradation by a group of low molecular mass proteases (30–65 kDa), plus one of 120 kDa [76]. In contrast, Coradi and Guimaraes in 2006 demonstrated that the hydrolysis of collagen type I by trophozoites lysates was associated with a broad enzymatic activity, from >116 to 18 kDa. [They used five strains isolated and axenized in Brazil and the reference strain Portland 1.] In all strains, the major proteolysis zones were visualized at [90- to 18-kDa] region, mainly the bands detected at 66, 45, 30, and 18 kDa and a diffuse zone ranging from 35 to 18 kDa. Differences on the hydrolysis patterns were observed in relation to the *Giardia* trophozoite strain [38]. The significance of these differences in the enzymatic activity remains to be determined, and it would be interesting to identify if it correlates with strain virulence. A subsequent study showed that these proteases are in fact secreted by trophozoites, since excretory/secretory products display collagenolytic activity in the same molecular range, mainly the activities of 145, 96, and 82 kDa bands. Inhibition assays showed that the main proteolytic activity against collagen type I in excretory/secretory products is due to CP [39].

The fact that trophozoites contain and/or release collagenases could be of special importance in giardiasis pathogenesis, particularly when it comes to alterations in the intestinal epithelium. Additional research is required to confirm this hypothesis, from the identification of the genes encoding for these collagenases to the use of animal models to test their contribution to the infection.

### 3.3. *Acanthamoeba* spp

*Acanthamoeba *is a free-living amoeba and is an opportunistic protozoan parasite. It is ubiquitously distributed throughout the environment. *Acanthamoeba *spp. are able to cause several diseases in humans, which are associated with immunocompromised patients in the case of granulomatous amoebic encephalitis and with contact lens wearers in the case of keratitis. More than 30 cases of *Acanthamoeba keratitis* were identified recently from the Chicago (Illinois) area alone. It is estimated that as of August 2006 more than 5000 cases of Acanthamoeba keratitis have occurred in the United States. Because Acanthamoeba keratitis is not a reportable disease in the United States, the actual number is not known and may be even higher. Large numbers of cases have also been reported from the United Kingdom and India [77].

The name of this protozoan comes from the presence of spine-like structures on its surface. This amoeba has a simple life cycle with two stages, a vegetative stage, or trophozoite, and a resistant stage, or cyst. 

Parasite adhesion to target cells or tissues is a necessary step to invade the host; this step is mediated by a 130 kDa mannose-binding protein (MBP), which is a surface-expressed protein [78]. Other adhesins include a laminin-binding protein of 28.2 kDa [79] and a 55 kDa protein that was found to bind to laminin in the pathogenic strain *A. culbertsoni *[80]. Furthermore, *A. polyphaga *binds to the ECM proteins collagen type IV, laminin and fibronectin [81], and calcium enhances this binding [82]. In these interactions, amoebas exhibit a stronger attachment to the basal membrane components laminin and collagen IV. The adhesion to these molecules leads to secondary responses, such as phagocytosis and toxin production, that result in host cell death via the phosphatidylinositol 3-kinase (PI3K) pathway [83]. Additionally, *Acanthamoeba *has been shown to display plasminogen activator activity, which can trigger host MMP leading to the degradation of basement membranes.* Acanthamoeba *also possesses hydrolytic enzymes, such as elastases [84], phospholipases [85], serine proteases [86–89], CP [86, 89], and contact metalloproteases [90].

There are many proteases in *Acanthamoeba *that are able to degrade certain components of ECM proteins (Table 1, Figure 1).

He et al. [91] described the presence of a collagenolytic enzyme that digested collagen shields and purified collagen *in vitro*. Collagen is one of the major components of the cornea, so keratitis is directly linked to the collagenolytic activity. More importantly, *in vivo* studies demonstrated the pathogenic features of this parasite product, as *A. castellanii-*conditioned medium produced lesions that resembled amoebic keratitis. The use of nonspecific protease inhibitors and ethylenediaminetetraacetic acid-Na (EDTA-Na) with *Acanthamoeba*-conditioned medium completely blocked the degradation of collagen shields, and the use of EDTA-Na *in vivo* also blocked amoebic collagenase activity. 

Mitro et al. [88] also described the collagenolytic activity of *A. polyphaga*-conditioned medium on the substrates Azocoll and gelatin (both denatured type I collagen) and native collagen type I. They concluded that *A. polyphaga *secretes multiple proteases of the serine, cysteine, and metalloprotease types and that all the proteases can contribute to the collagenolytic effect.

 Kong et al. [43] described the purification of a secretory serine protease of *A. healyi. *The purified protease had a molecular weight of 33 kDa, a pH optimum of 8.0, and a temperature optimum of 40°C. This protease degrades collagens type I and IV and fibronectin. The protease activity is inhibited by phenylmethylsulfonyl fluoride (PMSF) and diisopropylfluorophosphate (DIFP) serine protease inhibitors.

 Na et al. [41] purified a secreted protease from *A*.* castellanii *of approximately 12 kDa in molecular weight. This molecule was a chymotrypsin-like serine protease that could degrade various protein substrates, such as collagen, fibronectin, laminin, secretory IgA, IgG, plasminogen, fibrinogen, hemoglobin, and rabbit corneal protein. The researchers also used the purified protein to test cytopathogenicity toward HEp2 cells, which resulted in the loss of viability within 12 h. The cytopathogenic events were completely inhibited when the protease was pre-treated with PMSF before being added to the HEp2 cells.

 Kim et al. [44] purified a serine protease secreted by *A*. *lugdunensis.* The purified 33 kDa protease had a pH optimum of 8.5 and a temperature optimum of 37°C. This protease is able to degrade collagens type I and IV, fibronectin, fibrinogen, hemoglobin, albumin, IgG, and IgA. The use of PMSF inhibited almost all of the protease activity. Furthermore, Kim et al. [92] reported that this 33 kDa protease could be purified from different *Acanthamoeba *strains with different degrees of virulence. 

Sissons et al. [42] identified two proteases of 130 and 150 kDa from an *Acanthamoeba *isolate capable of inducing granulomatous encephalitis. The 130 kDa protease was inhibited by PMSF, suggesting that it is a serine protease, whereas the 150-kDa protease was inhibited by 1, 10-phenanthroline, suggesting that it is a metalloprotease. Both proteases exhibited maximal activity at neutral pH and over a range of temperatures. These proteases degrade ECM components, such as collagen I and III (major components of collagenous ECM), elastin, and plasminogen as well as casein and hemoglobin. 

 Ferreira et al. [93] characterized secreted elastase activities in the conditioned medium of *Acanthamoeba polyphaga*. These activities are in the range of 70–130 kDa, and they have an optimal pH of 7.5; additionally, they are inhibited by PMSF, antipain, chymostatin, and 1, 10-phenanthroline, and partially reduced by elastinal and EDTA. This study demonstrates that amoebic trophozoites secrete elastase activities and suggests the high-molecular-weight serine proteases as possible elastase candidates.

Finally, de Souza Carvalho et al. [40] described the partial biochemical characterization of extracellular proteolytic enzymes secreted by *Acanthamoeba *spp. trophozoites isolated from corneal tissue. Different enzymatic patterns of collagenases were observed, varying between single and multiple collagenase activities. Low-molecular-weight serine proteases were secreted by the trophozoites and were associated with a more severe clinical course of the keratitis. Consequently, *Acanthamoeba *proteolytic enzymes could be related to the degree of virulence and clinical manifestations of disease in human keratitis.

More studies are necessary to comprehend the importance of the proteases of this parasite in the diseases caused by *Acanthamoeba* spp. and also to design protease inhibitors as drugs to target *Acanthamoebic *proteases. 

### 3.4. *Naegleria* spp

*Naegleria *spp. are free-living amoebae that are found worldwide in warm fresh water and that feed mostly on bacteria. *Naegleria *spp. are amoeboflagellates that could transform from the trophozoite form into a flagellate if nutrients are limited. The amoebas can also transform into cysts to survive adverse conditions [94]. Species of *Naegleria *have been known for over a century [95], but it was only approximately 40 years ago that one species, called *Naegleria fowleri*, was found to cause primary amoebic meningoencephalitis (PAM) in human [96]. *N. fowleri *is a pathogen with a worldwide distribution; because the organism lives and multiplies in warm water, most cases of PAM occur in tropical regions. There are only 235 reported PAM cases worldwide, so the disease is rare. However, it is almost always fatal, with only approximately 5% of patients surviving, and it affects mostly children [94]. PAM affects the central nervous system (CNS), progresses rapidly, and is commonly fatal. In experimental animals, the amoebae gain access to the CNS by crossing the olfactory bulbs [97, 98]. Once there, the trophozoites divide rapidly and cause inflammation associated with tissue destruction, leading to death in a few days. The pathogenic mechanisms involved in the tissue invasion and destruction are poorly understood. However, various *in vitro* studies suggest the presence of many virulence factors that could be involved in the pathogenesis of PAM. These factors include the presence of adhesins [99], pore-forming proteins [100, 101], phospholipases [102], contact-dependent lysis [103], elastase [84], and secreted proteases with cytopathic effects [45, 104]. 

There are few reports concerning the adhesion of *N*. *fowleri* to ECM proteins. Han et al. [99] reported that *N. fowleri *possesses an integrin-like molecule that binds to immobilized fibronectin. This protein was described as being an *α*-integrin subunit and has a role in cytotoxicity. Shibayama et al. [105] described the interaction of *N. fowleri* with human collagen I. Recently, Jamerson et al. [106] compared the adhesion to collagen and fibronectin by the pathogenic *N. fowleri *strain and the nonpathogenic *N. lovaniensis, *finding greater adherence of *N. fowleri *to fibronectin. Cervantes-Sandoval et al. [107] found several differences between pathogenic *N. fowleri *and nonpathogenic *N. gruberi *in the expression of mannose and fucose glycoconjugates. *N. fowleri *presents higher levels of surface glycoconjugates that contain *α*-D-glucose and terminal *α*-L-fucose residues than *N. gruberi*. Cytosolic and membrane glycoconjugates showed greater expression in *N. fowleri *than in *N. gruberi*. These differences could be related to the adherence to different substrates, and, therefore, they could also be related to the pathogenesis of *N. fowleri*.

 Aldape et al. [45] partially purified a secreted protease activity of 30 kDa with two isoforms (Table 1, Figure 1). The biochemical properties of these two forms of *N*. *fowleri *protease activity were indistinguishable, suggesting that they might be posttranslationally modified isoforms of the same gene product. This activity was abolished by *trans*-Epoxysuccinyl-leucylamido(4-guanidino)butane (E-64) and leupeptin, cysteine protease inhibitors. Trophozoites or secreted protease activities were able to degrade mainly collagen and elastin ECM proteins; this effect was inhibited by ZFA-FMK, a specific cysteine protease inhibitor. Serrano-Luna et al. [104] described proteolytic activities from *N. fowleri *and *N. gruberi *that are able to degrade Azocoll at 37°C. These activities were mainly inhibited by cysteine protease inhibitors. More studies are needed to elucidate whether specific proteases from *N. fowleri *can degrade specific ECM proteins, such as collagens type I and IV, fibronectin, elastin, and laminin. 

The study of *Naegleria *virulence factors is still scarce; therefore, many studies have to be done in the future pointing out especially to the role of amoebic proteases in the invasion to the CNS. It is also necessary to develop new drugs against this parasite, and some of these drugs could target mainly CPs. 

### 3.5. *Trichomonas vaginalis*

*T. vaginalis* is a flagellated protist that is responsible for the most prevalent nonviral sexually transmitted infection (STI), with an annual estimate of 174 million new infections worldwide [108]. The parasite is capable of causing severe vaginal, ectocervical, prostatic, and urethral inflammations, and it is linked with sterility, pelvic inflammatory disease, adverse pregnancy outcomes, postnatal complications, and cervical cancers [109–113]. Furthermore, *T. vaginalis* also contributes to the HIV pandemic, along with other STIs, by boosting the efficiency of virus transmission [109, 111, 114, 115].

Cystic stages are unknown for *T. vaginalis*. The trophozoite attaches to the mucosal surfaces of the lower urogenital tract and divides by longitudinal binary fission. *T. vaginalis* survives long term in the varying and adverse acidic environment of the vagina through various successful mechanisms [116]. After cytoadherence, *T. vaginalis* transforms to an amoeboid structure with increased cell-to-cell surface contact, forming cytoplasmic projections that interdigitate with target cells. The interactions of *T. vaginalis* with mucins, vaginal epithelial cells, and ECM molecules persist in a non-self-limiting fashion [116]. 

The parasite readily attaches to surfaces with immobilized fibronectin and binds to fibronectin in a highly specific receptor-mediated fashion [117]. Interestingly, the enzyme glyceraldehyde 3-phosphate dehydrogenase (GAPDH) was found to be a surface-associated fibronectin-binding protein of *T. vaginalis*. GAPDH was upregulated by iron; accordingly, higher levels of binding to FN were observed for organisms grown in an iron-replete medium. GAPDH is not involved in the cytoadherence of trichomonads, but it binds collagen [118]. Unknown surface proteins and carbohydrates appear to mediate parasite binding to immobilized laminin. Just as happened with fibronectin, *T. vaginalis* adhesion proteins that mediate cytoadherence were found not to be involved in laminin binding [117, 119].

*T. vaginalis* encodes an impressive repertoire of candidate proteases, with almost 450 genes [120], making *T. vaginalis* one of the richest protease-containing protozoans in nature [120, 121]. An* in silico* search for possible surface-bound candidates to degrade ECM molecules showed that in the genome draft, 122 *T. vaginalis* entries are transmembrane proteases (TPs). These proteases are better known in the human system, where they fulfill multiple functions, including degrading ECM proteins and cell-cell and cell-ECM adhesion, and are thought to be important in neoplastic, inflammatory, and infection sites [121, 122]. There are also 53 *T. vaginalis* glycoprotein 63-like sequences (GP63). GP63 in *Leishmania* are involved in binding to host cells and degradation of various host proteins, including proteins from the immune system and ECM proteins [121, 123].

In addition to these *in silico* inferred proteases that are possible candidates to degrade ECM, there are three reports of *T. vaginalis* CP activities degrading components of the ECM: CP30, CP39, and CP65. These data are summarized in Table 1 and Figure 1 [46–48]. 

The CP30 fraction was obtained by performing a binding assay of total *T. vaginalis* proteins to fixed Hela cells and then collecting the eluted proteins. These Hela-binding proteins are able to degrade collagen IV and fibronectin, but not laminin 1, in the region corresponding to 30 kDa, by the zymogram technique [46]. Using a gelatin two-dimensional (2D) zymogram, the researchers determined that the protease activity belonged to a cysteine protease, as it was inhibited by E-64, and they detected two spots in this MW region; however, this fraction was not tested again with ECM substrates. Using a polyclonal antibody raised against the entire 30 kDa Hela-binding fraction separated by 1D gels, they located the CP30 fraction at the *T. vaginalis* surface and in the cytoplasm; they also inhibited *T. vaginalis* adhesion to Hela cells [46]. Furthermore, *T. vaginalis* isolates with low levels of cytoadherence had little or none of the 30 kDa protease activity [124]. These data suggested a relationship between the CP30 fraction with proteolytic activity and cytoadherence. The researchers also found that the CP30 fraction is immunogenic and is secreted by *T. vaginalis  in vitro* (culture media) and *in vivo* (vaginal washes). Interestingly, parasite cells grown in contact with Hela cells appear to release higher levels of the CP30 fraction [46]. Because the researchers were working with a fraction, it is not possible to know whether a single protein is responsible for all the detected activities: gelatinase, collagenase, fibronectinase, immunogen, adhesin, surface protein, cytosolic protein, and secreted protein. It is important to emphasize that CP30 was active on collagen IV and fibronectin only at a pH of 4.5 and 5.0; beyond this pH, no CP30 activity was detected, indicating that the *in vitro* optimal conditions for CP30 activity are consistent with the environmental conditions found in the urogenital tract of women. For example, the vaginal pH in healthy women ranges from 4.0 to 5.0 and in women with ongoing trichomoniasis from 4.4 to 7.0 [46, 125]. Thus, CP30 could degrade certain ECM proteins in the first step of infection, when the vaginal microenvironment is acidic.

The CP39 fraction was studied using the same strategy and showed almost the same behavior as the CP30 fraction, with the exception that more substrates were tested, and it was found that this fraction degrades collagen I, II, and V in addition to collagen IV and fibronectin [47]. The 39 kDa protease band is formed by only one spot in a 2D gelatin zymogram, with an MW of 37.5 kDa and a pI of 4.9; the protein was identified by mass spectrometry. *TvCP39* had the motifs typical of a novel clan CA, family C1, cathepsin L-like CPs [126, 127]. The antibody against the purified recombinant protein did not recognize the original 37.5 kDa protease in total protease-rich extracts. Instead, it recognized two spots of 28 and 24 kDa with pI 5.0, which were identified by mass spectrometry as part of the *TvCP39* cytotoxic protease. The authors concluded that the antibody cannot identify the mature protease, probably due to posttranslational modifications such as N-linked glycans. Using this antibody, it was observed that *TvCP39* is located on the surface of the parasite and is secreted during active infection [47, 127] supporting the role of *TvCP39* as a potential biomarker for trichomoniasis [126] in vaginal secretions. Additionally, *TvCP39r* binds to the surface of Hela cells and protects them from trichomonal cytotoxicity, probably by competing with the native *TvCP39 *for the binding sites on Hela cells.

 Sommer et al. [128] showed that the CP30 fraction composed of *TvCP4*, *TvCP39, * and, in smaller proportion, TvCP3 was able to induce apoptosis in human vaginal epithelial cells (HVECs). The initiation of apoptosis is correlated with protease activity, as the specific cysteine protease inhibitor E-64 inhibited both activities [128]. Whether the mechanism involved in the cellular damage by *TvCP39* is through induction of programmed cell death as was identified for the entire CP30 fraction requires further investigation [126]. 

The CP65 fraction was studied using the same strategy as for the CP30 and CP39 fractions, and it showed almost the same behavior, degrading collagen IV and fibronectin [48]. Subsequently, they determined the proteolytic activity and the corresponding protein pattern in 2D gel electrophoresis to identify the TvCP65 protein spot and the coding partial gene [129]. The partial sequence was identified as a typical clan CA, family C1, and cathepsin L-like CP. The antibody against the purified recombinant protein recognized TvCP65 in total lysates of *T. vaginalis* and on the parasite surface. The antibody also inhibited *T. vaginalis* induction of cytotoxicity. The recombinant fragment of CP65 binds HelA cells and prevents the native CP65 binding [129].

In the case of TvCP65, the partial gene was identified previous to the release of the *T. vaginalis* genome draft, so the entire gene was not obtained. Remarkably, a recent study of the *T. vaginalis* degradome also showed, in the 2D zymogram, proteolytic activity in the 63–70 kDa regions, which might be related to this previously described TvCP65 protease. The CPs identified suggested that these high-MW spots are formed by two strongly bonded CP with MWs between 34.6 and 33.7 kDa, that are resistant to the denaturing and reducing conditions used during the 2D procedure [120, 126, 130]. 

All three fractions identified genes coalescing in some way in the 30 kDa region, which is in agreement with the findings in the *T. vaginalis* degradome; most of the 27 proteolytic spots detected in 2D zymograms are encoded by only nine distinct genes identified with theoretical MWs in the 30 kDa region (*TvCP1*, *TvCP2*, *TvCP3*, *TvCP4*, *TvCP4-like*, *TvCP12*, *TvCPT*, *TvLEGU-1*, and another legumain-like cysteine protease) [126]. Therefore, there may be three different proteases that are actually within the same MW range of 30 kDa and that behave differently in zymograms because of their different processing stages, posttranslational modifications, or dimerization. Alternatively, the signals may all correspond to the same protease, and further research would clarify this question. Moreover, after the protease genes of fractions CP30, CP39, and CP65 were identified, the ability to degrade ECM proteins was not tested for each one, so it remains to be determined which one of these proteases is responsible for ECM protein degradation.

Because the secreted fractions CP30 [46], CP 39 [47], and CP65 [48] were able to degrade several types of collagens, they might also be the molecules involved in the cervical softening observed before labor [131], or preterm labor in women with trichomoniasis [47, 48, 132, 133]. Further research should be performed to corroborate the role of such proteases in the tissue damage that occurs during trichomoniasis. 

### 3.6. *Trypanosoma brucei*

*T. brucei* is a protozoan parasite responsible for thousands of infections every year of African trypanosomiasis, with two variants: in animals, the disease is known as nagana, and in humans it is known as sleeping sickness or human African trypanosomiasis (HAT). This disease is widespread throughout the African continent. The transmission vectors are the tsetse flies that inoculate *T. brucei* parasites into the blood of its mammal host. Trypanosomiasis presents two stages: first, trypanosomes are observed in the hemolymphatic system, producing fever, splenomegaly, adenopathies, endocrine disarrays, and cardiac and neurological or psychological disorders. In this stage, trypanosomes multiply rapidly, infecting the spleen, liver, lymph nodes, skin, heart, eyes, and endocrine system. In the later stage, trypanosomes are distributed in the CNS, leading to several sensory, motor, and psychic disorders, and culminating in death [134, 135].

To reach the inner tissues in its host, the parasite *T. brucei* secretes proteases into the ECM (Table 1, Figure 1), such as the 40 kDa neutral metalloprotease that permits the parasite to move and migrate by degrading collagen, fibronectin, and laminin [49]. This activity is inhibited by EDTA, ethylene glycol tetraacetic acid (EGTA), phenanthroline, and tetracycline [134]. The GP63 zinc metalloprotease, the most important matrix metalloproteinase (MMP) in the parasite, is a surface enzyme that was first reported in *Leishmania*. This protein is highly conserved among species in terms of homology. This enzyme performs several functions in different stages of the trypanosome life cycle, and the development of specific inhibitors provides new treatments for this parasitic disease [50].

In the later stages of the disease, when the trypanosomes cross the blood-brain barrier (BBB), the extracellular release of metalloproteases and cell adhesion molecules from *T. brucei* contributes to the BBB disruption by the modification of the ECM components, and these molecules can be used as markers for early diagnosis of the disease progression from the first to the second stage. This information is important because the treatment differs between the two stages and is more complicated in the case of the later stage of the disease [136]. 

A prolyl oligopeptidase gene (POPTc) homolog in *T. brucei* has been identified, POPTb, and the secondary structure has been obtained. Recombinant POPTb shows a structural composition similar to POP from *T. cruzi* and similar sensitivity to inhibitors. This enzyme is able to degrade collagen, contributing to pathogenesis [56].

Associated proteases participate in the process of traversal across the BBB, as the *T. brucei* CP, brucipain, and cathepsin B (*Tb*CatB). Brucipain induces calcium activation signals that open up the barrier, allowing parasite crossing. *Tb*CatB is upregulated *in vivo*, suggesting the participation of this protein in the parasite internalization. CP can activate a class of G protein-coupled receptors (GPCRs) known as protease-activated receptors, or PARs. The activation of PARs increases the BBB permeability. The participation of PAR-2 in a calcium-mediated signaling pathway allows the trypanosomes to cross into the CNS [17, 52]. 

Gene-specific RNAi can be induced in bloodstream parasites in an experimental model of trypanosome infection. Induction of RNAi targeting *Tb*CatB transcripts, led to reduced protease activity *in vivo* rescuing mice from a lethal *T. brucei* infection, as it was observed in previous *in vitro* RNAi experiments. In the murine model of infection, trypanosomes expressing *Tb*CatB RNAi did not present splenomegaly, and parasites were not detected in blood, due to the inability of parasites to effectively enter into other tissues. This constitutes an important evidence of the role of *T. brucei* proteases in the degradation of ECM proteins and also in the colonization and invasion of different organs in the host [53]. 

## 4. Intracellular Protozoa

### 4.1. *Trypanosoma cruzi*

This protozoan parasite causes human Chagas disease, a chronic and debilitating condition affecting 10 million people from Mexico to Argentina and Chile. *T. cruzi *is transmitted either by an insect vector that has access to the host via breaches in the skin or through mucosal membranes, mainly the conjunctival or gastric mucosa. It is an obligate intracellular parasite that disseminates from the initial infection sites to the heart and smooth muscle, with several rounds of invasion, growth, and egress from infected cells during the acute infection. Very little is known regarding the early interactions between the parasite and its host that facilitate the establishment of the infection [137]. 

*T. cruzi* is also transmitted through blood transfusion, organ transplantation, ingestion of contaminated food or fluids, and congenital or sexual transmission [138]. Vertical transmission of *T. cruzi* cannot be prevented, but with early detection and treatment it can be cured with 100% success [139]. During congenital *T. cruzi* infection, the parasite reaches the fetus by crossing the placental barrier. The placentas from women infected with *T. cruzi* exhibit severe alterations in the ECM. This result provides evidence that the parasite induces reorganization of the ECM in a way that regulates the inflammatory and immune responses of the host. In this context, the parasite load and the immunological status of both mother and fetus, which influence the probability of congenital transmission of *T. cruzi,* are determinants for the infection [140]. In the infective process, collagen, heparan sulfate, and laminin are destroyed by the parasite, but interestingly, fibronectin is not affected, so the selective destruction of the ECM could be part of the invasion mechanism [140]. 

At the site of primary infection, the metacyclic trypomastigotes infect local macrophages, fibroblasts, and mesenchymal tissues, but the infection of distant tissues after dissemination through the blood vessels is unknown. Several pieces of evidence have shown that *T. cruzi* interacts with host ECM components, not only producing the breakdown products that play an important role in parasite mobilization and infectivity but also altering the presence of cytokines and chemokines, allowing the escape of the parasite from the inflammatory and immune responses [140]. 

During tissue invasion, *T. cruzi* interacts with different elements of the ECM, facilitating the internalization into different cells in the underlying connective tissue [141]. Adhesion is very important for the parasite, which presents various surface molecules, such as the GP85 fibronectin receptor [142] and GP83, that bind to human cells to regulate the expression of laminin, needed to enter the host cell [143, 144]. These glycoproteins that bind to collagen, laminin, and fibronectin allow the parasite to permeate and migrate into the ECM barrier. A recent study of the human ECM interactome of *T. cruzi* and its GP83 ligand shows that this interaction is important for understanding the molecular pathogenesis of the infection and could lead to novel approaches to intervention in Chagas disease [144].

**Figure 2:**
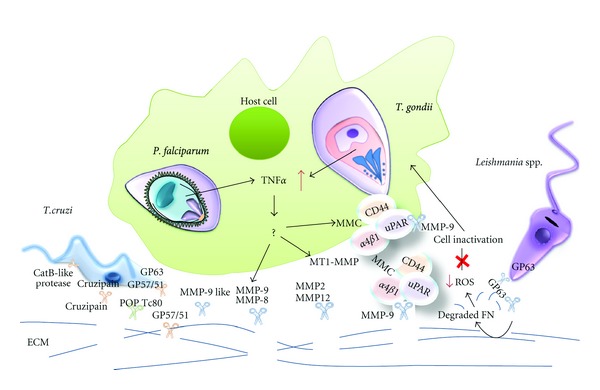
Intracellular parasite proteases. CPs: cysteine proteases, pink scissors; SPs: serine proteases, green scissors; MMPs: matrix metalloproteases, blue scissors; ECM extracellular matrix; POP: prolyl oligopeptidase; CatB: cathepsin B; MMC: migratory molecular complex; ROS: reactive oxygen species.

A prerequisite for host cell invasion is that *T. cruzi* must cross the ECM barriers. Through mechanisms that are not well understood, the parasite induces the expression of ECM molecules or decreases their presence. The more obvious explanation for the decrease of ECM is that the parasite destroys the ECM by the secretion of proteases. Several products with characteristics of proteases were studied in this parasite; they include CPs, serine proteases, and metalloproteases (Table 2, Figure 2).

GP57/51, cruzain or cruzipain, a cysteine protease of the papain family, is the best characterized protein in *T. cruzi*. It is synthesized during all developmental stages of *T. cruzi*, but in a regulated manner, and amastigotes and trypomastigotes contain 10-fold lower levels than epimastigotes [158]. The enzyme is present in lysosomes and reservosomes, and certain isoforms are associated with the plasma membrane, whereas others are secreted into the medium and are capable of degrading collagen, fibronectin and highly antigenic proteases [147, 159, 160]. The crystal structure of the protein shows a unique active site feature, which suggests that the design of specific inhibitors could reduce parasitemia and infection with no effect on mammalian cells [161]. Cruzipain is inhibited by organomercurial reagents such as E-64, tosyl-L-lysinechloromethyl ketone (TLCK), and cystatins, such as peptidyl diazomethane [159, 162], or by the 2,3,5,6,-tetrafluorophenoxymethyl ketone inhibitor, which totally eliminates *T. cruzi *parasites. Thus, specific inhibitors have a high potential as novel antiparasitic agents [163]. Cruzipain is structured as one catalytic domain, with high sequence identity with cathepsin S, and a long C-terminal domain, characteristic of the CP in trypanosomatids. The mature enzyme is encoded by several arranged genes containing repeated units encoding the pre-proenzyme form with the C-terminal extension [160, 164]. Previous studies have demonstrated that infection can be treated in cell, mouse and dog models by the inhibition of cruzipain [165, 166].

GP63, or penetrin is a surface protease that promotes adhesion to heparin, heparan sulfate, and collagen. This molecule could play a very important role in host cell invasion after migration through the ECM. It is localized on the surface, promoting the selective adhesion of trypomastigotes in a saturable way and promoting adhesion and spreading of fibroblasts [145]. Although it has not been determined whether this protease degrades ECM proteins, it is very important for *T. cruzi* binding to the ECM and for host cell invasion.

POPTc80 serine protease is a member of the prolyl oligopeptidase family (POP). It catalyzes the cleavage of several ECM components, such as collagen types I and IV and fibronectin [154] and is localized inside a vesicular compartment close to the flagellar pocket, which suggests that its secretion and local action on ECM components are required for infection. Specific protease inhibitors blocked parasite entry into the cells [167].

Matrix metalloprotease-9-like (MMP-9-like) activity is an extracellular metalloprotease released by *T. cruzi*. It acts as a regulator of parasite infection and pathogenesis of Chagas disease, with a molecular mass of 97 kDa in cellular extract and an 85 kDa polypeptide in both cellular and secreted parasite extracts. These proteins were recognized by an anti-MMP-9 polyclonal antibody that localized them on the surface of *T. cruzi. *Doxycycline, which exhibits direct MMP-9-inhibiting properties *in vitro*, inhibited these MMP-9-like activities. This ECM-degrading enzyme is important for the parasite-host interaction [152]. MMPs of the family of zinc-dependent peptidases that regulate ECM-eukaryotic cell interactions can be involved in normal matrix remodeling or pathological tissue destruction. The gelatinases MMP-2 and MMP-9 are important in many physiological and pathological processes in mammals.

30 kDa cathepsin B-like protease is another cysteine protease identified and produced in all forms of *T. cruzi* parasites that degrades human type I collagen. Its N-terminal sequence shows high similarity to cathepsin B protease [150]. It is a glycoprotein localized in the reservosomes [160].

Pathogenoproteomics is the study of the interactions among host, vector, and parasite, which aims to understand infections with particular attention to the proteases in the secretome of trypanosomes as important molecules for virulence and pathogenicity [168], just as CPs are known to play an indispensable role in the biology of parasitic organisms [169] and suspected to act as a major pathogenic factors in mammalian hosts.

Specific interactions between *T. cruzi* and ECM components play an important role in parasite distribution, mediating basement membrane and ECM degradation as well as adhesion to and invasion of host cells. The ECM-binding sites on the *T. cruzi* surface could be potential therapeutic targets by inhibiting the parasite spreading. 

The complete genome of *T. cruzi* is still unknown [170], and several proteases have been identified, although most of them have not been biochemically well characterized. Cruzipain is the best characterized protease, and it has been proposed as a virulence factor in Chagas disease [171] due to its participation in the invasion of mammalian cells. In this regard, the treatment of *T. cruzi*-infected mice with specific protease inhibitors resulted in their effective rescue from lethal infection, and parasitological cure of most of them. This effect was observed even in an immunodeficient murine model [172]. These results are very hopeful, since they clearly indicate that proteases could be considered as valid targets for chemotherapy in Chagas disease. In fact, efforts to develop new drugs for chemotherapy have been recently shown to be effective for the treatment of Chagas disease in animal models [173].

### 4.2. *Leishmania*

*Leishmania *are kinetoplastid dimorphic protozoan parasites of vertebrate macrophages that cause the chronic sandfly-borne disease leishmaniasis. It is estimated that 1.5 to 2 million children and adults develop symptomatic disease each year, resulting in more than 70,000 deaths (primarily from visceral leishmaniasis) and an infection prevalence of 12 million people [174]. Different species of *Leishmania* are responsible for a spectrum of human diseases, ranging from the self-healing cutaneous forms caused by *L. major, L. tropica*, and *L. mexicana* to the more severe mucocutaneous disease caused by *L. braziliensis* and finally to the most severe form, the visceral disease caused by *L. donovani* [175].

*Leishmania* develops within the midgut of the sandfly vector as flagellated promastigote stages that transform through a number of physiological states, culminating in the nondividing, metacyclic promastigotes that are preadapted for life in the mammalian host. Metacyclic promastigotes are injected into the skin when female sandflies take a blood meal and are phagocytosed by a variety of host cells, including neutrophils, dendritic cells, and macrophages that are equipped to clear invading microbes. However, internalized promastigotes differentiate into nonflagellated amastigotes that can replicate within lysosome-like compartments, or parasitophorous vacuoles, within these cells [176]. *Leishmania* surviving intracellularly produce multiple effects in phagocytes, including inhibition of the respiratory burst, prevention of apoptosis, inhibition of chemotaxis in both macrophages and neutrophils, and suppression of the Th1-type protective response [177]. 

In addition, during the intracellular life of *Leishmania,* this protozoan requires a repertoire of adaptations to assure entry-exit from the cell as well as to thwart innate immune mechanisms and prevent clearance. These adaptations include the invasion and destruction of host tissues and the penetration of host vascular systems, enabling the parasites to migrate to sites specific for their growth and development. Concerning the interplay between *Leishmania* species and ECM, several studies suggest that this interaction occurs through protease secretion and expression of ECM-binding proteins on the surface of the parasite [156, 157, 178–180].

Ghosh et al. [180] identified, isolated, and characterized an *L. donovani* promastigote surface protein that binds with high affinity (Kd in the nanomolar range) to laminin, a major adhesive glycoprotein of the ECM and basement membrane. In addition, a prominent laminin-binding protein of 67 kDa was identified on the promastigote surface. In the process of tissue invasion, there is likely an association of the parasite with the host epithelial cell surface via a receptor-adhesion-like interaction. Importantly, several authors have indicated that ECM components provide a mechanism of adherence for different human pathogens, such as *Candida albicans*, *Paracoccidioides brasiliensis,* and trichomonads, that express laminin-receptor-like molecules that mediate cellular attachment to eukaryotic host cells [180].

Concerning proteolytic activity against the ECM, several researchers reported the degradation of collagen and fibronectin by promastigotes of *L. amazonensis. *Importantly (Table 2, Figure 2), McGwire [157] found that promastigote migration through the ECM is enhanced by a 63 kDa glycoprotein, a zinc-dependent metalloprotease (syn. GP63 or leishmanolysin). They used a Matrigel assay, where approximately 40% of the GP63 expressing promastigotes had migrated into the lower chamber at 12 h after inoculation, while only 7% of GP63-deficient had migrated at the same time. Additionally, purified leishmanial GP63 from stationary-phase promastigotes was effective in digesting collagen type IV and fibronectin. After incubation with GP63, it began a digestion of the proteins into smaller units that became a smear of smaller proteins of less than 15 kDa. Interestingly, the patterns of digested fibronectin observed by SDS-PAGE differed somewhat depending on the source of GP63 used, and cell-associated GP63 appeared to digest fibronectin into larger subunits than did purify GP63. Finally, laminin appeared to be resistant to digestion by GP63, as it remained intact as protein subunits regardless of the conditions used for incubation. In addition, when GP63 was inactivated by preincubation with a zinc chelator, orthophenanthroline, this metalloprotease did not degrade fibronectin [157]. Importantly, it was found that leishmanolysin is able to facilitate complement inactivation in serum [181], participating in the interaction with the host macrophages and in intraphagolysosomal survival [179, 182]. 

Following this line of research, Kulkarni et al. [156] showed that both promastigotes and amastigotes of *Leishmania* species (*L. amazonensis, L. major, L. donovani*) can bind directly to soluble fibronectin and laminin and that promastigotes express a distinct surface protein of ~60 kDa that binds both ECM proteins. The results presented strongly indicate that the protein(s) that bind fibronectin and laminin are distinct from leishmanolysin. Because fibronectin and laminin bound to parasite surface proteins of nearly identical molecular weights, it is likely that they may bind the same surface receptor. Importantly, a rapid and extensive surface proteolytic degradation of fibronectin by promastigotes of multiple *Leishmania* species was found. Fibronectin was cleaved into 10 to 13 fragments that ranged in size from 240 to 25 kDa, and complete degradation occurred by 24 h for all parasite lines. Additionally, *Leishmania*-degraded FN decreased the production of reactive oxygen intermediates by parasite-infected macrophages and affected the accumulation of intracellular parasites. The authors suggest that the binding of FN and laminin via this receptor may increase the proximity of surface-localized leishmanolysin to FN, resulting in its enhanced degradation. These results support the idea that cutaneous *Leishmania* species express a receptor protein functionally analogous to the microbial surface component recognizing adhesive ECM molecules. Furthermore, multiple *Leishmania* species can extensively degrade FN in a rapid manner using surface leishmanolysin, which suggests that this process is functionally conserved and may contribute to the pathogenesis of different forms of leishmaniasis. It is likely that the binding of ECM proteins, such as FN, to the cell surface receptor may lead to signal transduction within parasites, resulting in changes in gene expression that facilitate further parasite invasion or stage transformation [156]. 

Some studies reported the participation of ECM domains that may be potentially important for the activity of macrophages in innate immunity. Interestingly, Kulkarni [156] found several fragments that encompassed nearly the entire FN protein being degraded at the extreme N- and C-terminal ends. Smaller fragments of ~60 and 25 kDa were each composed of two and three comigrating fragments of the same size, respectively. One of the 60 kDa fragments encompassed the region of FN containing the RGD domain, and the 28 and 25 kDa fragments overlap and encompass the FN ICS domain. It is possible that the proteolytic degradation of FN by *Leishmania* may expose this region for interaction with macrophages in these assays and that the interaction of macrophages with this or other FN fragments may lead to their deactivation.

Currently, only a limited number of drugs are available for treating severe cases of cutaneous, mucocutaneous, and visceral leishmaniasis, although none is optimal due to their toxicity or teratogenicity, expense, requirements for hospitalization, and/or the widespread emergence of drug resistance [183–185]. As an alternative strategy, vaccination is also in experimental and clinical trials [186]. There is still great potential for the discovery and design of potent inhibitors that selectively target GP63 to block or reduce *Leishmania* infection by favoring the functional activation of the macrophage. In the case of the intracellular *Leishmania *parasite, the amastigote stage may selectively take up inhibitors. Small molecule protease inhibitors might mimic amino acids or purines for which the parasite has a specific uptake mechanism. Furthermore, homologous host proteases are generally present in lysosomes, a less accessible subcompartment within mammalian cells [187].

Because the ultimate goal of invading *Leishmania* is to become intracellular, McGwire [157] proposes that enhanced migration at the site of inoculation may promote parasite binding to and phagocytosis by macrophages. Furthermore, migration through the ECM and basement membrane may facilitate the access of parasites to the blood or lymph circulation for dissemination to distant sites [178, 180], where they may parasitize tissue macrophages [157]. Supporting this hypothesis, GP63-deficient parasites have shown to have diminished virulence in mice [188, 189]. However, other GP63-dependent events may account for these results. In fact, many different roles have been assigned to this protein, such as (i) evasion of complement-mediated lysis, (ii) facilitation of macrophage phagocytosis of promastigotes, (iii) inhibition of natural killer cellular functions, (iv) resistance to killing by antimicrobial peptide, (v) degradation of macrophage and fibroblast cytosolic proteins, and (vi) promotion of survival of intracellular amastigotes [190]. The multiple functions of this protein make difficult the assessment of ECM degradation impact in the parasite virulence. Therefore, additional studies are necessary, using more controlled conditions, such as mice expressing collagen and FN mutated in the cleavage site of GP63, where we can dissect only the ECM degradation role of GP63. Additional research would be the use of green-fluorescent protein (GFP) and GP63-deficient *Leishmania* to precisely track them when invading at the beginning of the infection, instead of late infection measures such as parasite burden or lesion sizes which reflect more complex phenomena [188, 189, 191].

### 4.3. *Toxoplasma gondii*

Toxoplasmosis is caused by *T. gondii*, an obligate intracellular protozoan [192]. This parasite has a worldwide distribution and is considered to be one of the most successful on earth [192, 193]. The tissue cyst-forming coccidium *T. gondii* can probably infect all warm-blooded animals (mammals and birds) and humans, with the cat being the only definitive host. Although up to one third of the human world population is infected with *T. gondii* [194], most infections are asymptomatic. Primary infection is usually subclinical, but in some patients, cervical lymphadenopathy or ocular disease can be present. Infection acquired during pregnancy may cause severe damage to the fetus. In immunocompromised patients, reactivation of latent disease can cause life-threatening encephalitis [192, 194].

Within the feline intestinal epithelium, the parasites go through a sexual cycle, resulting in oocyst shedding [195]. In its intermediate hosts, such as humans, the parasites go through a sexual cycle, and infection is mainly acquired by ingestion of food or water contaminated with oocysts or by eating undercooked or raw meat containing tissue cysts [194]. The wall of these cysts is digested inside the host stomach, and the released bradyzoites will invade the small intestine. Within the small intestine, they transform into tachyzoites, the rapidly growing, disease-causing form. Tachyzoites, which can infect most nucleated cells, replicate inside a parasitophorous vacuole and egress, leading to cell death and rapid dissemination to neighboring cells [196]. A strong inflammatory response causes the clinical manifestations of infection. Tachyzoites transform into bradyzoites under the pressure of the host immune system. This slowly replicating form of the parasite resides inside cysts that localize mainly in the skeletal muscle and the brain for the life of the host [197–199].

A hallmark of *T. gondii* infections is passage of parasites across restrictive biological barriers-intestine, BBB, blood-retina barrier, and placenta during primary infection or reactivation of chronic disease. Traversal of cellular barriers permits the rapid dissemination of parasites to gain access to biologically restricted organs. This process involves active parasite motility and tightly regulated interactions between host cell receptors and parasite adhesins that facilitate paracellular transfer. Infected murine macrophages express less alpha4 and alpha5 integrin and are less adhesive to FN, laminin, or collagen during early infection [200], and adoptively transferred infected immature dendritic cells (DCs) show diminished expression of beta2 integrin [201]. Thus, *T. gondii* may alter the adhesive interactions of leukocytes, evade the host immune system, and disseminate to immunoprivileged sites [200], suggesting that parasites use murine macrophages [200], monocytes [202], and DCs [201] as “Trojan horses” to disseminate in the organism while avoiding immune attack [203]. Moreover, to reach these immunoprotected sites, *T. gondii* must control how these “Trojan horses” degrade the ECM proteins potentially with proteases such as MMPs [204].

Infection of murine macrophages from the cell lineage Raw 264.7 with the RH strain produced an increase in cellular migration through a 3D matrix (Matrigel), and the presence of MMP inhibitor I drastically decreased the migration [205]. This observation demonstrates *in vitro* how *T. gondii* induces the macrophages' machinery of invasion to achieve dissemination using MMPs (Table 3, Figure 2).

Surprisingly, it was demonstrated that *T. gondii* (RH strain) infected human monocytic cells (THP-1) have decreased proMMP-9 (progelatinase B) secretion and expression [204]. MMP-9 is a secreted metalloprotease that is central in the migratory molecular complex, suggesting that this metalloprotease is fundamental for the migration of infected macrophages [205]. Opposite to what Bauche [204] observed, Seipel [205] observed that *T. gondii* infection increased the secretion of an active MMP-9 form [214, 215]. Confirming what Seipel [205] reported, recent research has shown that *T. gondii* GPIs induce the production of MMP-9 in human macrophage-like THP-1 cells via a TLR2/4 in a TNF*α*-dependent mechanism [206].

The secretion of MMP-9 requires an intermediary step, through docking at the cell surface. Shed CD44, serine urokinase-type plasminogen activator receptor (uPAR), and *α*4*β*1 or *α*v*β*3 integrins form a complex at the cell surface and function as a docking structure for proMMP-9. In cancer metastasis, these molecules are often secreted as a multiprotein complex [216, 217]. Further experiments demonstrate the presence of soluble CD44, suggesting that *T. gondii* promotes shedding of CD44, mediating the secretion of MMP-9 [205], as observed in other pathological and physiological conditions [217, 218]. Schuindt [219] showed in RH-infected Raw 264.7 macrophages by immunoprecipitation assays that MMP-9, CD44 TIMP-1, and uPAR were secreted as a multiprotein complex by infected macrophages. These data suggest that similar events to those observed in metastatic cells might take place during macrophage harboring of *T. gondii* [219].

The major physiological activators of proMMP-2 (progelatinase A) are members of the MT-MMP (membrane-type MMP) family, and for MT1-MMP, this process involves the action of tissue inhibitor of matrix metalloprotease (TIMP-2). TIMP-2 (a physiological proMMP-2 inhibitor) forms a complex with active MT1-MMP that serves as a cell surface ‘‘receptor” for proMMP-2. *T. gondii*-infected THP-1 exhibited a decrease in both proMMP-2 and TIMP-2. However, *T. gondii* infection did promote the expression and accumulation of a 60 kDa active form of MT1-MMP [204, 205]. MT1-MMP was originally identified as an activator of MMP-2 and was later shown to degrade various ECM components, including collagen types I, II, and III, FN, laminin, and proteoglycans [208]. Moreover, it was demonstrated that proteolysis of the ECM by MT1-MMP stimulates focal adhesion turnover, which regulates integrin-generated signal transduction and subsequent cell migration [220, 221]. MT1-MMP [222] is also involved in cell-cell and cell-matrix interactions and CD44 shedding, along with ADAM10 [223, 224]. ADAM10 is increased in *T. gondii*-infected macrophages [205].

Integrin *α*v*β*3 is fundamental for crossing the BBB, and it is also able to regulate the binding of *α*2*β*1 to FN and the conversion of pro-*α*v to the mature *α*v subunit. This conversion is achieved by MT1-MMP in breast carcinoma cells [222] but is usually performed by proprotein convertases (PCs) [225]. Just as occurs in carcinoma cells, the MT1-MMP pathway might be a preferential pathway for processing prointegrin subunits in *T. gondii*-infected macrophages [205]. Furthermore, *T. gondii* metalloproteases could also play a role in processing prointegrin subunits [205]. 

There are few *in vivo* studies on this topic, and they have shown that MMP-2 [209] and MMP-9 [226] are elevated in the intestine of *T. gondii*-infected mice. It was shown that IL-23 is essential in the development of small intestinal immunopathology by inducing local MMP-2 upregulation [209]. Additionally, using Knockout (KO) mice and inhibitors, it was demonstrated that MMP-2 but not MMP-9 is an essential downstream mediator of immunopathology in *T*.* gondii*–induced ileitis [209], suggesting that MMPs could be involved in tissue remodeling/repair, at the small intestine during *T*. *gondii* peroral infection [226]. In a recent clinical study, increased concentrations of MMP-12 and elastin degradation products were detected in the serum of pregnant women infected with *T*.* gondii*. Co-immunoprecipitation of MMP-12 with elastin suggested that MMP-12 might mediate the pathological degradation of elastin in pregnant women with toxoplasmosis [210, 227].

This is an exciting new field for *T. gondii* and for other parasites [227], and the utilization by intracellular parasites of part of the migratory molecular complex appears to be a common practice observed in several physiological and pathological mechanisms involving migration, which may facilitate the access of infected leukocytes to immunoprivileged sites in the host [204, 205].

### 4.4. *Plasmodium falciparum*

This parasite produces the illness named malaria and affects one million people every year, with the most vulnerable population being children under 5 years of age in Africa [108]. The parasite is widespread in tropical and subtropical regions, including much of sub-Saharan Africa, Asia, and the Americas. Malaria is a mosquito-borne infectious disease of humans that results from the multiplication of *Plasmodium *parasites, first within hepatocytes, resulting in tens of thousands of parasites that burst from the hepatocyte. Individual organisms then invade red blood cells and undergo an additional round of multiplication. Parasites inside red blood cells cause symptoms that typically include fever and headache, in severe cases progressing to coma or death.

The mechanisms leading to severe malaria, of adhesion and release of bioactive products, are not entirely understood. Once malaria sporozoites enter the bloodstream, they infect hepatocytes, where they are able to replicate extensively. The ability of the parasite to arrive and colonize the liver is directed by two proteins, the circumsporozoite (CS) protein and the thrombospondin-related adhesive protein (TRAP), which recognizes the heparan sulfate proteoglycans and thrombospondin in the ECM, respectively. The parasite uses these proteins to obtain entry into the liver parenchyma [228]. 

As was previously reported, phagocytosis of trophozoite/hemozoin by adherent human monocytes stimulates the production of TNF-*α* and other proinflammatory cytokines, inducing the synthesis of MMPs. These molecules degrade matrix proteins and disrupt the basal lamina [212]. 

Once parasite adhesion is established, several host-derived enzymes, such as MMP-9 and TIMP-2, increased in patients with severe malaria [212]. Other studies have revealed an increase in MMP-8 with no difference in MMP-9 levels and the participation of the TIMP-1 and -2 [213]. Therefore, MMPs and TIMPs are involved in the pathogenesis of malaria (Table 3, Figure 2). MMPs are important for the disease and the resolution phases of acute and chronic inflammatory processes, facilitating entry into the tissues [213]. 

In *Toxoplasma* and *Plasmodium* parasites, MMP-9 activation is a specific step for trophozoite/hemozoin-fed monocytes, it is dependent on TNF-*α* production and is inhibited by using anti-TNF-*α* antibodies or by the pharmacological inhibition of this protease [212]. 

Host-derived enzymes induced by parasites may cause immunopathological conditions that could be relevant in the pathogenesis of malaria and toxoplasmosis, either as proteolytic enzymes that degrade the ECM or as effectors and regulators of the immune response [212, 213]. The participation of these proteases has already been described in different inflammatory diseases such as bacterial meningitis, sepsis, tuberculosis, multiple sclerosis, and BBB dysfunction [229]. In this regard, studies on MMP KO animals, human genetic, and epigenetic as well as biochemical studies using natural or synthetic inhibitors of these MMP will provide a better understanding of the pathophysiology of these parasitic diseases [229]. 

One example is the therapeutic potential of the antibodies produced against the activated forms of MMP-2 and -9 has been reported in murine models of inflammatory bowel disease. The dysregulated MMPs are targets for the inhibitory antibodies in a resembling way when TIMPs were used, with the consequent diminishment of the infection [230]. Another strategy is the use of specific inhibitors of MMP to produce brain damage attenuation in infant rats after pneumococcal meningitis [212]. 

To increase the understanding of the specific role of these proteases, MMPs deficient (KO) mice have been used [211, 231]. Interestingly, in these studies, an important equilibrium between proteolytic and anti-proteolytic activity of MMPs, was observed besides, the possibility to find an effect on the proteolytic compensation by different MMPs or other classes of proteases. These results confirmed the necessity of further investigation to elucidate the role of MMP in infectious diseases.

Traditional physiological functions of MMPs were the modulation and regulation of ECM, but, presently, these proteases have been related with the disease development. Their participation in cancer metastasis, chronic inflammation, and tissue damage has permitted to establish that MMPs contribute to the generation of protein species with hugely differing activities [229].

MMPs and TIMPs impact disease development and, therefore, could be relevant as future targets for adjuvant intervention and offer a new chance to control pathogenic mechanisms in malaria and toxoplasmosis.

## 5. Concluding Remarks

The host-parasite relationship is a complex phenomenon that is mediated by virulence factors from the parasite as well as exacerbated responses from the host. Parasite destruction of ECM might involve the participation of many types of protozoan parasite proteases: CPs, serine proteases, and MMPs. For many of these parasites, the identity of the ECM proteases is unknown, because the reports refer only to proteolytic activities of certain molecular weight ranges. Therefore, it is important to identify each gene responsible for such proteolytic activity to have a better understanding of the parasite pathogenesis. 

Once the gene has been identified, it is important to use parasites in which protease genes are deleted, or overexpressed. This will be of great value to elucidate the actual role of parasite proteases as virulence factors in migration and invasion of the host tissues. Parasites migrating through interstitial tissue or a basement membrane are confronted with three-dimensional tissue structures of complex and varied physicochemical properties [232]. In this context, it is necessary to challenge parasite protease activities using *in vitro *and *in vivo* models of complex substrates such as 3D synthetic ECM (Matrigel) [233] as *in vitro* assays or isolated endothelial basement membranes [234], collagen-rich interstitial tissue [235], and provisional wound matrix as examples of *in vivo* models [235].

On the other hand, the participation of host cells in the invasion and migration of parasites is of relevant importance, because they can be persuaded by parasites like *E. histolytica* and *Acanthamoeba* to increase the production of MMPs. Furthermore, host cells like the macrophages are used by intracellular parasites, like *T. gondii* and *Plasmodium*, as “Trojan horses” to invade tissues in a way that resembles metastatic behavior of cancer cells. 

The study of proteases and their inhibitors is relevant to the search for new therapeutic targets or treatment strategies, or to improve the early diagnosis of human parasitic diseases and increase the power of the drugs used in treating these diseases.

## Figures and Tables

**Table 1:** Extracellular parasite-derived proteases that degrade human ECM proteins.

Parasite	Protease	Purified	Gene	Type	ECM Substrates				Localization	Reference
					Collagen	Fibronectin	Laminin	Elastin
*Entamoeba histolytica*	72 kDa protease	No	ND	ND	I	ND	ND	ND	Crude extracts EDG	[33]
	Major neutral protease (56 kDa)	Yes	ND	CP	I	Yes	Yes	ND	ND	[21]
	EhCP112	Yes	ND	CP	I	Yes	ND	ND	Secreted	[34]
	117, 97, and 68 kDa	No	ND	ND	I	ND	ND	ND	EDG	[35]
	EhCP5	Yes	Yes	CP	I	ND	ND	ND	Secreted	[36, 37]
*Giardia intestinalis*	66, 45, 30, and 18 kDa	No	ND	CP	I	ND	ND	ND	Crude extracts	[38]
	145, 96, and 82 kDa	No	ND	CP	I	ND	ND	ND	Secreted	[39]
*Acanthamoeba *spp.	70–130 kDa	No	ND	SP	ND	ND	ND	Yes	Secreted	[40]
*Acanthamoeba castellanii*	12 kDa	Yes	Yes	SP	I	Yes	Yes	ND	Secreted	[41]
	130 kDa	No	ND	SP	I, III	NO	NO	Yes	Secreted	[42]
	150 kDa	No	ND	MMP	I, III	NO	No	Yes	Secreted	[42]
*Acanthamoeba healyi *	33 kDa	Yes	ND	SP	I, IV	Yes	ND	ND	Secreted	[43]
*Acanthamoeba lugdunensis*	33 kDa	Yes	ND	SP	IV	Yes	Yes	ND	Secreted	[44]
*Naegleria fowleri*	30 kDa	Yes	ND	CP	I	ND	ND	Yes	Secreted	[45]
*Trichomonas vaginalis*	CP30	No	ND	CP	IV	Yes	No	ND	Cytoplasm	[46]
									Cell surface	
									Secreted	
	CP39	No	ND	CP	I, III, IV, V	Yes	ND	ND	Secreted	[47]
									Cytoplasm	
									Cell surface	
	CP65	No	ND	CP	IV	Yes	No	ND	Cytoplasm	[48]
									Cell surface	
*Trypanosoma brucei*	40 kDa neutral metalloprotease	Yes	ND	MMP	Yes	Yes	Yes	ND	Secreted	[49]
	GP63 zinc metalloprotease	Yes	Yes	MMP	I	ND	ND	ND	Cell surface	[50, 51]
	Brucipain	Yes	Yes	CP	I	ND	ND	Yes	Lysosome	[52–55]
	Cathepsin B (tbCatB)	Yes	Yes	CP	I	ND	ND	ND	Lysosome	[54]
	POP Tb	Yes	Yes	SP	Yes	ND	ND	ND	Released into the host blood stream	[56]

CPs: cysteine proteases; SPs: serine proteases; ECM: extracellular matrix; ND: not determined; EDG: electron-dense granules; MMPs: matrix metalloproteases; POP: prolyl oligopeptidase.

**Table 2:** Intracellular parasite-derived proteases that degrade human ECM proteins.

Parasite	Protease	Purified	Gene	Type	ECM substrates				Localization	Reference
					Collagen	Fibronectin	Laminin	Elastin
*Trypanosoma cruzi*	GP63 or penetrin	Yes	Yes	ND	ND	Yes	Yes	ND	Cell surface	[145, 146]
	Cruzipain	Yes	Yes	CP	Yes	ND	ND	ND	Reservosome, cell surface, and secreted	[147–149]
	Cathepsin B-like protease	Yes	Yes	CP	I	ND	ND	ND	Lysosome	[150, 151]
	MMP-9 -like proteases (116.1 to 101.3 kDa)	Yes	ND	CP	IV, V, XI, XIV	ND	Yes	Yes	Secreted cell surface	[152, 153]
	POP Tc80	Yes	No	CP	I, IV	Yes	ND	ND	Secreted	[154, 155]
*Leishmania*	GP63 or leishmanolysin	Yes	Yes	MMP	IV	Yes	No	ND	Cell surface and secreted	[156, 157]

**Table 3:** Intracellular parasite induced proteases that degrade human ECM proteins.

Parasite	Protease	Type	ECM substrates				Localization	Reference
			Collagen	Fibronectin	Laminin	Elastin
*Toxoplasma gondii*	MMP-9 or gelatinase B	MMP	IV, V, XI, XIV	ND	Yes	Yes	Infected macrophage surface and secreted	[153, 205–207]
	MT1-MMP or MMP-14	MT-MMP	I, II, III	Yes	Yes	ND	Infected macrophage surface	[153, 204, 205, 208]
	MMP-2 or gelatinase A	MMP	I, II, III, IV, V, VII, X, XI	Yes	Yes	Yes	Secreted by unknown host cell	[153, 207, 209]
	MMP-12 or macrophage elastase	MMP	I, IV, V	Yes	Yes	Yes	Secreted by unknown host cell	[153, 207, 210, 211]
*Plasmodium falciparum*	MMP-9 or gelatinase B	MMP	IV, V, XI, XIV	ND	Yes	Yes	Secreted by unknown host cell	[153, 212]
	MMP-8	MMP	I, II, III	ND	ND	ND	Secreted by unknown host cell	[153, 213]

MT1-MMP: membrane-type MMP.
